# Enhancement of the Influenza A Hemagglutinin (HA)-Mediated Cell-Cell Fusion and Virus Entry by the Viral Neuraminidase (NA)

**DOI:** 10.1371/journal.pone.0008495

**Published:** 2009-12-30

**Authors:** Bin Su, Sébastien Wurtzer, Marie-Anne Rameix-Welti, Dominic Dwyer, Sylvie van der Werf, Nadia Naffakh, François Clavel, Béatrice Labrosse

**Affiliations:** 1 Inserm U941, Génétique et Ecologie des Virus, Institut Universitaire d'Hématologie, Hôpital Saint-Louis, Université Paris Diderot-Paris 7, Paris, France; 2 Institut Pasteur, Unité de Génétique Moléculaire des Virus à ARN, CNRS URA 3015, Université Paris Diderot-Paris 7, Paris, France; 3 Centre for Infectious Diseases and Microbiology, Institute of Clinical Pathology and Medical Research (ICPMR) Westmead Hospital, University of Sydney, Sydney, Australia; University of Minnesota, United States of America

## Abstract

**Background:**

The major role of the neuraminidase (NA) protein of influenza A virus is related to its sialidase activity, which disrupts the interaction between the envelope hemagglutin (HA) protein and the sialic acid receptors expressed at the surface of infected cells. This enzymatic activity is known to promote the release and spread of progeny viral particles following their production by infected cells, but a potential role of NA in earlier steps of the viral life cycle has never been clearly demonstrated. In this study we have examined the impact of NA expression on influenza HA-mediated viral membrane fusion and virion infectivity.

**Methodology/Principal Findings:**

The role of NA in the early stages of influenza virus replication was examined using a cell-cell fusion assay that mimics HA-mediated membrane fusion, and a virion infectivity assay using HIV-based pseudoparticles expressing influenza HA and/or NA proteins. In the cell-cell fusion assay, which bypasses the endocytocytosis step that is characteristic of influenza virus entry, we found that in proper HA maturation conditions, NA clearly enhanced fusion in a dose-dependent manner. Similarly, expression of NA at the surface of pseudoparticles significantly enhanced virion infectivity. Further experiments using exogeneous soluble NA revealed that the most likely mechanism for enhancement of fusion and infectivity by NA was related to desialylation of virion-expressed HA.

**Conclusion/Significance:**

The NA protein of influenza A virus is not only required for virion release and spread but also plays a critical role in virion infectivity and HA-mediated membrane fusion.

## Introduction

Influenza A viruses are enveloped viruses expressing two major transmembrane glycoproteins incorporated into the viral envelope: the hemagglutinin (HA), and the neuraminidase (NA). Based on the antigenicity of hemagglutinin and neuraminidase, influenza A viruses are subdivided into 16 HA (H1-H16) and 9 NA (N1-N9) subtypes.

The HA glycoproteins are expressed as trimers at the surface of virions [1], [2] and mediate viral entry into target cells through recognition and binding to terminal sialic acid groups on membrane-bound proteins and lipids of the host cell. Most of these host cell receptors consist of terminal *N*-acetyl sialic acid (SA) linked to galactose or *N*-acetylglucosamine with an α2,6 linkage or with an α2,3 linkage [3], [4], [5]. This interaction leads to the internalization of the virus into an endosome whose acidic environment allows HA proteins to mediate fusion between the viral and endosomal membranes [6], [7], thereby delivering the viral genomic RNA segments into the cytoplasm of the target cell.

The NA proteins, organized on the surface of virions as tetramers, catalyse cleavage of terminal sialic acids from the viral receptors [8], [9]. The sialidase activity of neuraminidase is believed to promote liberation and dissemination of progeny viral particles from infected cells late in infection [10], to facilitate virus spread through the mucus overlying the human airway epithelium [11], and to restrict the rate of superinfection of influenza A target cells [12]. Surprisingly, in spite of the relative abundant expression of NA at the surface of influenza virions [13], a direct contribution of NA in the process of viral entry into target cells has never been clearly established. Some studies have suggested that NA could help virions reach putative sites of endocytosis located on the cell surface [14]. These studies, however, were based on the analysis of the ability of NA inhibitors to block viral entry into target cells that do not produce mucin, and provided contradictory results [7], [15].

In this study, we sought to determine the extent that NA could play a direct role in viral entry, and to delineate the impact of NA activity on virus adsorption, endocytosis, and membrane fusion. These potential effects were evaluated using two sensitive, quantitative and reproductible phenotypic assays: a pseudotype infectivity assay using HIV-derived virions expressing influenza A HA and NA proteins, and a cell-cell fusion assay. We found that the presence of NA significantly enhanced both pseudotype infectivity and cell-cell fusion, establishing that beyond its previously recognized functions, NA plays a direct and early role in influenza A virus entry into host target cells.

## Results

### Impact of trypsin treatment, acidic pH and NA expression on HA-mediated cell-cell fusion

**Figure 1:**
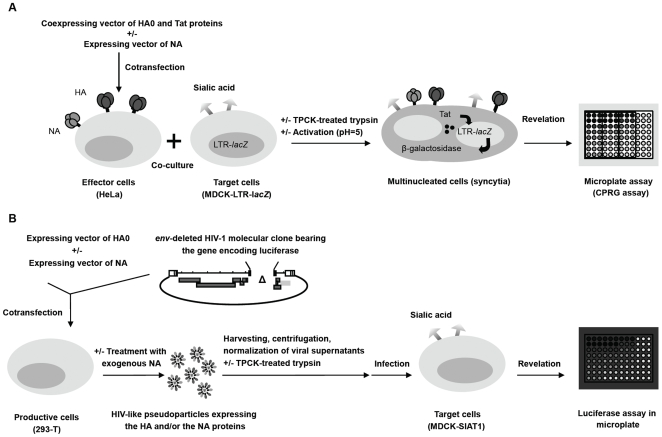
Schematic representations of the cell-cell fusion (panel A) and the infectivity assays (panel B). Panel A: Cell-cell fusion assay. HeLa cells seeded into 96-well plates were cotransfected with a plasmid coexpressing the HA and Tat proteins in the presence or in the absence of a vector expressing NA. Transfected cells were further co-cultured with MDCK target cells that harbor a Tat-inducible β-galactosidase reporter system (LTR-*lacZ* cassette). Co-culture was successively treated with TPCK-treated trypsin and with acidic medium to mediate membrane fusion. 40 hours later, cells were lysed and the β-galactosidase activity was detected using a colorimetric assay. Panel B: Infectivity assay. Subconfluent monolayer of 293-T cells were cotransfected with an *env-*defective HIV-1 proviral clone in which the *nef* gene was replaced by that encoding the *Renilla* luciferase and with plasmids expressing HA and/or NA. NA can also be provided in this system as exogenous and soluble NA from *Clostridium perfringens*. Viral supernatants were harvested, cleared, normalized by quantification of the HIV-1 p24 antigen content and further used to infect MDCK-SIAT1 target cells seeded into 96-well plates. Infected cells were incubated for 40 hours, and further lysed. Lysates were tested for luciferase activity by the addition of a specific substrate measured in a luminometer.

**Figure 2:**
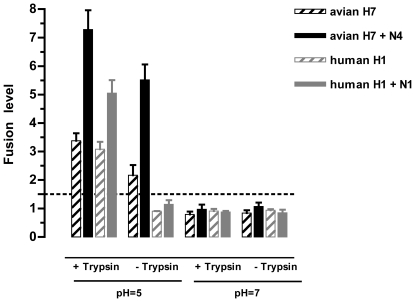
Validation of the cell-cell fusion assay. HeLa cells, which were transfected with a plasmid co-expressing both HA and Tat proteins, in the absence or in the presence of the plasmid encoding the NA protein, were co-cultured with the MDCK-LTR-*lacZ* target cells. Co-cultures were subsequently transiently treated, if any, with a medium containing TPCK-treated trypsine to cleave the polyprotein precursor HA0 into the mature proteins HA1 and HA2, and further with an acidic medium to trigger membrane fusion mediated by the envelope proteins. The HA and NA proteins tested derived either from the human H1N1 A/Paris/0650/04 virus or from a highly pathogenic avian H7N4 virus. The fusion level was defined as a ratio between the mean OD value obtained for each sample and that of the threshold control corresponding to the background noise due to the artefactual transactivation by Tat, and was considered positive when greater than 1.5 (dotted line). Columns represent mean fusion level value of at least three independent experiments (bars represent standard errors).

Cell-cell fusion assays have proven in the past to be highly useful for the study of entry of many viruses, including retroviruses, rhabdoviruses, herpesviruses, flaviviruses and filovirus [16], [17], [18], [19]. The cell-cell fusion assay used in this study (Figure 1A) required that two important conditions be fulfilled. First, fusion mediated by influenza HA can only occur after cleavage of the HA0 precursor into its two mature subunits HA1 and HA2, a process that admits two alternative configurations. When the precursor carries a monobasic cleavage site, which is the case in most influenza A strains, HA0 is cleaved on the cell surface during natural infection by extracellular trypsin-like proteinase [20]. To reproduce this phenomenon, and because HeLa cells do not express these proteinases, the co-cultures are transiently treated with a trypsin preparation in which chymotrypsin activity has been blocked by TPCK treatment. In other influenza A strains, such as subtypes H5 and H7 from highly pathogenic viruses, the HA0 precursor carries a polybasic proteolytic site that is cleaved intracellularly in the trans-Golgi compartment by proteinases of the subtilisin family [20], [21]. In this case, no exogenous proteinase treatment is necessary to mediate membrane fusion. Second, entry of influenza A viruses occurs in endosomes, where the acidic environment induces HA conformational changes that control its fusogenic properties. Therefore, our cell-cell fusion assay also required that the pH of the coculture medium be transiently lowered to pH 5.0 to induce membrane fusion. To evaluate these two aspects, we tested the fusogenic properties of two HA proteins, one from a highly pathogenic H7N4 avian influenza strain and one from a primary H1N1 human strain (Figure 2). We first tested the importance of the maturation of the HA proteins to induce fusion. As expected, the H1 protein, which is characterized by a monobasic cleavage site, required an exogenous trypsin treatment (at a final concentration of 5 µg/mL for 15 min) to induce significant fusion rate of 3.1. On the other hand, the rates of fusion induced by the H7 protein, which has an intact polybasic cleavage site (ERKRKR) that can be targeted by ubiquitous intracellular host proteases, were of 2.2 and 3.4 in the absence and in the presence of exogenous enzymatic treatment, respectively. We then determined the pH dependence of the fusion process. Not surpringly, cell fusion was only obtained when cocultures were transiently treated with acidic buffer at pH 5.0, while no fusion was observed at neutral pH (7.0).

We next explored the effect of the presence of NA on HA-mediated fusion. To do so, we cotransfected the plasmids encoding the homologous HA and NA proteins with a DNA plasmid ratio (w/w) of 1∶2 (Figure 2). In these conditions, fusion mediated by the H1 protein was increased 1.6-fold (*p* = 0.0199, t test) by N1, while H7-mediated fusion was increased 2.5-fold (*p* = 0.0068) and 2.2-fold (*p* = 0.058) by N4, in the absence and in the presence of TPCK-treated trypsin treatment, respectively. These results demonstrate that NA exerts a direct enhancement of HA-mediated fusion.

### Enhancement of HA-mediated fusion by NA is dose-dependent and requires NA enzymatic activity

**Figure 3:**
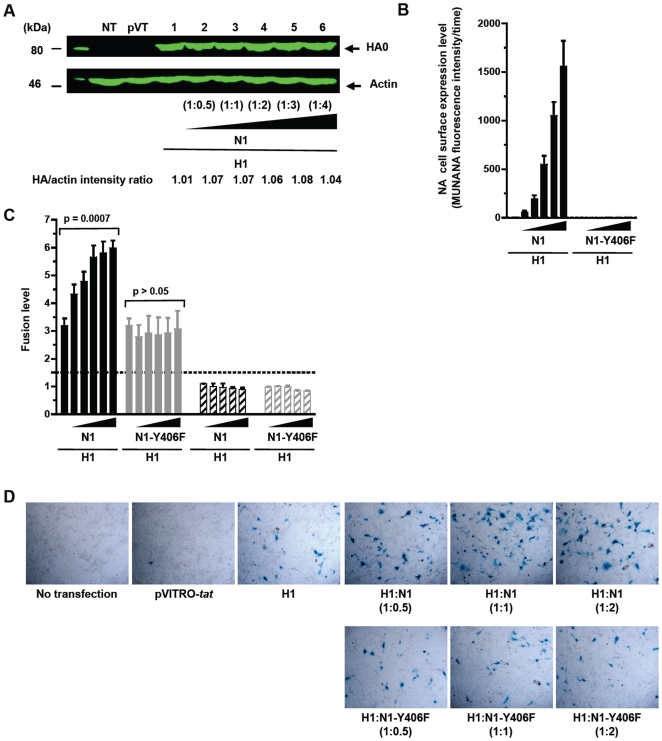
Efficiency of HA-mediated membrane fusion was dependent of NA enzymatic activity. HeLa cells were transfected with different ratios (w/w) of a plasmid expressing the H1 protein from the H1N1 A/Paris/0650/04 virus and a plasmid encoding either the wild-type homologous N1 protein or a defective enzymatic variant of this protein, N1-Y406F. The HA:NA plasmid ratios (w/w) tested were: 1∶0, 1∶0.5, 1∶1, 1∶2, 1∶3 and 1∶4. The co-cultures, which were performed with the MDCK-LTR-*lacZ* target cells, were successively treated with TPCK-treated trypsine and acidic medium (pH 5.0). Panel A: quantification of HA0 in transfected HeLa cells by western blot. Results are representative of two independent experiments. Panel B: indirect quantification of surface expression of NA by measurement of its enzymatic activity using the MUNANA substrate. Panel C: as described in the legend of Figure 2, the fusion level was defined as a ratio whose threshold of significativity is represented by a dotted line on the graph. Columns represent mean fusion level value of at least three independent experiments (bars represent standard errors). Panel D: *in situ* X-Gal staining of cocultures.

We next evaluated the impact of increasing HA:NA plasmid ratios (w/w) on the rates of cell-cell fusion (Figure 3). In these experiments, we used the H1 protein from the A/Paris/0650/04 strain, in combination with the homologous N1 protein. The range of HA:NA ratios extended from 1∶0 to 1∶4. As described above, these experiments were conducted following treatment with trypsin and with acidic medium (pH 5.0). To make sure that coexpression of NA did not affect expression of HA, cotransfected HeLa cell extracts were analysed by western blot for HA expression. No variations in HA0 expression were seen as a result of increasing amounts of coexpressed NA (Figure 3A). In parallel, surface expression of NA was evaluated by an enzymatic assay using the small substrate MUNANA (Sigma) at a high concentration (100 µM). As expected (Figure 3B) NA activity increased linearly with the amount of NA-expressing plasmid in the cotransfection.

As shown on figure 3C, we observed again a significant increase in fusion when HA was co-expressed with NA. This increase was clearly dose dependent (*p* = 0.0007, Kruskal-Wallis test), suggesting that this process is directly dependent on the level of the NA activity. It is noteworthy, however, that while a significant increase in fusion was seen between 1∶0.5 and 1∶2 (*p* = 0.0343, t-test) the enhancement of fusion by NA was clearly less pronounced and appeared to plateau between 1∶2 and 1∶4. To test the importance of NA enzymatic activity in this phenomenon, HA was cotransfected with a defective enzymatic variant of the N1 protein carrying a key aminoacid substitution in the active site at position 406, N1-Y406F. With this mutant, no effect of NA on fusion was seen at either HA:NA ratio (*p*>0.05, Kruskal-Wallis test), demonstrating that the impact of NA on HA-mediated fusion is directly related to the sialidase activity of NA.

In parallel experiments, cell-cell fusion was evaluated by quantifying the number of cells expressing β-galactosidase using *in situ* X-Gal staining and microscopic examination. These experiments confirmed the results presented above, with a clear increase in the number and size of blue foci as a result of increasing the amounts of coexpressed NA (Figure 3D). The number of blue foci observed when the enzymatically inactive N1-Y406F was coexpressed with HA were not significantly different from those observed when HA was expressed alone. As expected, only very rare blue cells were observed in the absence of HA expression.

### Impact of NA on the infectivity of HA-expressing HIV-1 pseudotypes

**Figure 4:**
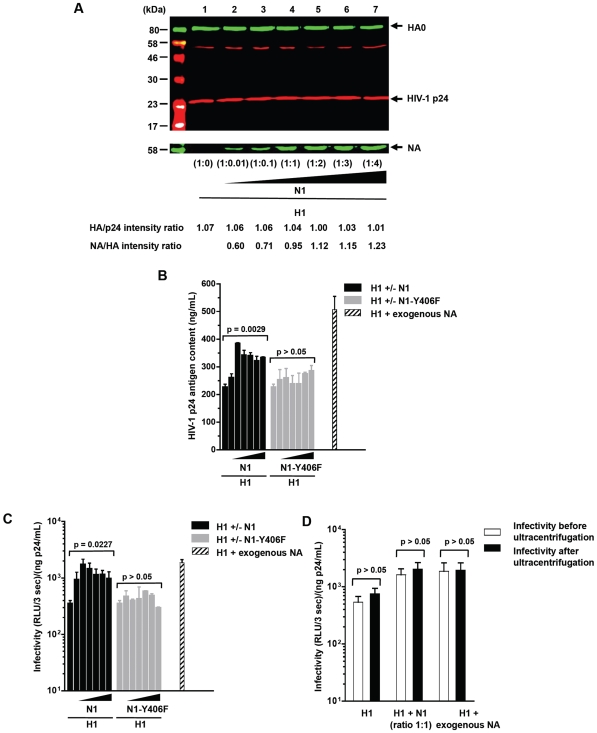
Infectivity of HA-expressing HIV-1 pseudotypes in the absence or in the presence of viral NA protein or soluble bacterial sialidase. 293-T cells were cotransfected with an *env*-defective HIV-1 proviral clone encoding *Renilla* luciferase and different ratios of a plasmid expressing the H1 protein from the H1N1 A/Paris/0650/04 virus and a plasmid encoding either the wild-type homologous N1 or a defective enzymatic variant, N1-Y406F. The HA:NA plasmid ratios (w/w) tested were: 1∶0, 1∶0.01, 1∶0.1, 1∶1, 1∶2, 1∶3 and 1∶4. Panel A: quantification of HA and NA expression in purified virions preparations by western blot. Results are representative of two independent experiments. Panel B: impact of NA activity on the production and release of pseudoparticles measured by HIV-1 p24 ELISA. Columns represent mean value of at least three independent experiments (bars represent standard errors). Panel C: infectivity of HA-expressing HIV-1 pseudotypes in the absence or in the presence of viral NA protein or soluble bacterial sialidase. Columns represent mean infectivity value of at least three independent experiments (bars represent standard errors). Panel D: infectivity of pseudoparticles expressing H1 alone, coexpressing H1 and N1 at a plasmid ratio of 1∶1, and expressing H1 alone but pretreated with neuraminidase from *Clostridium perfringens* prior to harvesting the virions. Columns represent mean infectivity value of at least three independent experiments (bars represent standard errors).

To confirm the impact of the NA protein on HA-mediated entry, we evaluated the extent that NA could also increase infectivity of HA-expressing HIV-1 pseudoparticles. Again, we used the H1 protein from the human influenza A strain H1N1 A/Paris/0650/04, in the absence or in the presence of the homologous N1 protein. Cultures of 293-T cells were cotransfected with the pNL4-3-ΔENV-LucR vector and ratios of plasmids encoding the HA or NA proteins ranging from 1∶0.01 to 1∶4. In these experiments, the amounts of transfected HA-encoding plasmid remained constant. Correspondingly, the amounts of HA0 incorporated into pseudoparticles, as measured by western blot analysis, were not affected by the increase in HA:NA plasmid ratio, while the amounts of particle-associated NA protein indeed increased (Figure 4A).

As expected the expression of NA resulted in a significant increase in the release of pseudoparticles (figure 4B), as measured by HIV-1 p24 ELISA (*p* = 0.0029, Kruskal-Wallis test), an effect that was not seen with the defective NA variant N1-Y406F (*p*>0.05). Similarly, the production of HA-expressing pseudoparticles was increased 2.2-fold when producer cells were pretreated with an exogenous NA from *Clostridium perfringens* (*p* = 0.0286, t-test).

The infectivity of each viral supernatant was measured on MDCK-SIAT1 cells after careful normalization of the amounts of pseudoparticles in the supernatants by HIV-1 p24 ELISA. MDCK-SIAT1 cells, which overproduce the α2,6-sialyltransferase enzyme, express twice the amounts of α2,6-linked sialic acids and two-fold less of the α2,3-linked sialic acids than parental MDCK cells [22]. As a result, MDCK-SIAT1 cells are clearly more susceptible to infection by human influenza A viruses than MDCK cells (data not shown).

Similar to what was seen with the cell-cell fusion assay, coexpression of N1 significantly increased the infectivity [(RLU/3 sec)/(ng p24/mL)] of H1-expressing pseudoparticles (*p* = 0.0227, Kruskal-Wallis test) (Figure 4C). Although this increase was clearly dose-dependent for the lowest H1:N1 ratios used, the effect of N1 rapidly reached a plateau, starting at the 1∶1 ratio. Not surprisingly, the infectivity of H1-expressing pseudoparticles was not affected when the defective N1-Y406F mutant was co-expressed, regardless of the plasmid ratios tested (*p*>0.05, Kruskal-Wallis test). These results again suggest that the potentialisation of the entry process mediated by the HA envelope proteins is related to the sialidase activity of NA, but that excess NA may interfere with this enhancement effect.

We then tested the possibility that exogenous neuraminidase could recapitulate the effects of influenza NA coexpressed with HA on the surface of pseudoparticles. In these experiments, we compared the infectivity of pseudoparticles expressing H1 alone, coexpressing H1 and N1 at a 1∶1 ratio, and expressing H1 alone but pretreated with purified exogenous neuraminidase from *Clostridium perfringens* (Figure 4D). As described above, coexpression of H1 and N1 at a 1∶1 ratio produced a 4-fold increase in infectivity. Interestingly, treatment of H1 pseudoparticles with exogenous neuraminidase produced a comparable effect. To test whether this increase in infectivity was the result of the activity of the enzyme on a target cell substrate or on a virion substrate, we compared the infectivity of neuraminidase-treated virions that had been separated from the enzyme by ultracentrifugation with that of viral suspensions that still contained bacterial neuraminidase. Interestingly, after normalization for HIV-1 p24 content, no difference in infectivity was seen between these two viral preparations, demonstrating that the NA-mediated infectivity increase in influenza HA pseudoparticles is the consequence of NA activity on a substrate that is expressed at the surface of virions, rather that at the surface of target cells.

## Discussion

Using a cell-cell fusion assay and an HIV-based pseudotype infectivity assay, we demonstrate here that NA expression can directly enhance HA-dependent influenza virus fusion and infectivity in a manner that is both dose-dependent and activity-dependent. Enhancement of fusion and infectivity increased with increasing the amount of NA protein at the surface of cells and of virions, until a plateau was reached. This effect was strictly dependent of the enzymatic activity of NA, as an active-site mutant of the enzyme (Y406F) had no impact on HA-mediated fusion and infectivity. Our results also strongly suggest that the impact of NA on infectivity and fusion was mediated by desialylation of residues expressed in virus producer cells or on virions, as opposed to desialylation of residues on target cells. Indeed, the extent that NA increased infectivity of pseudoparticles was comparable whether NA was coexpressed at the surface of virions or added to the pseudoparticle suspension as a soluble bacterial sialidase. In the latter case, enhancement of infectivity was detected even after ultracentrifugation and removal of soluble NA from the inoculum, thereby excluding an effect of NA on target cell sialic acid residues. This clearly rules out a non-specific enhancement through removal of repulsive negative charges from cell surfaces, similar to that described by Sun et al. [23], [24], where propagation and syncytium formation by HIV-1, a virus that does not express NA, was enhanced by sialidase treatment of the infected cultures.

Several virological functions have been assigned to the sialidase activity of influenza NA. First, it has been known for some time that NA activity facilitates the release of progeny virions from infected cells by cleaving the interactions between HA proteins and terminal sialic acid expressed on the cell surface or on adjacent virions. Indeed, efficient propagation in cell culture of mutant viruses lacking sialidase activity requires addition of large amounts of exogenous soluble neuraminidase to the medium [10]. Second, the NA protein is able to cleave sialic acid residues in mucus and cellular glycocalyx, a process that is believed to facilitate diffusion and dissemination of the virus within the respiratory tract, giving influenza virions larger access to target cells of the airway epithelium [11]. Third, it was also proposed that the NA protein could restrict the rate of superinfection of influenza A target cells by removing surface sialic acid receptors, thereby limiting the emergence of reassortant virus variants [12]. The data presented in our study now establish that neuraminidase can exert a direct enhancing effect on influenza infectivity and membrane fusion.

While the mechanism through which NA can achieve such an enhancing effect was not fully resolved here, our observations are consistent with the possibility that NA facilitates HA-dependent fusion by removing HA-bound sialic acid residues from the virus-producing cell. The removal of sialic acid residues from HA by NA could enhance fusion and infectivity following three mechanisms. First, NA could act on glycoconjugates carried by various lipids and proteins expressed at the cell surface whose terminal sialic acid residues can interfere with the receptor binding site of HA spikes. This unmasking could facilitate later binding of HA to sialic acid receptors expressed at the surface of target cells. Second, the desialylation of HA by NA could involve acid sialic residues carried by the HA glycoprotein itself and surrounding the receptor-binding site of HA, a site that is protected from neutralization by abundant glycosylation [25]. Again, partial unmasking of this region by desialylation could facilitate further binding of HA to the sialic acid residues expressed on surface of target cells, thus enhancing HA-mediated fusion. Finally, desialylation of HA could facilitate proteolytic cleavage of the HA0 precusor into its two mature and functional subunits HA1 and HA2, as suggested by observations that removal of N-glycosylation sites in close vicinity of the HA0 cleavage site can modulate its access to cellular proteases [26]. For most influenza hemagglutinins, this cleavage event occurs at the cell surface and could possibly be fully completed at the surface of virions, a property that is consistent with our observation that exogeneous sialidase treatment enhances the infectivity of HA-expressing pseudoparticles.

In our experiments, the enhancement of HA-mediated membrane fusion by NA was more prominent in the infectivity assay than in the fusion assay. Correspondingly, the HA:NA ratio at which enhancement reached a plateau was lower in the infectivity assay than in the fusion assay. These differences could relate to the number of HA trimer complexes that are competent for the formation of a fusion pore, which may differ at the surface of a cell expressing and at the surface of a virion. They could also relate to the different cell types used in the two assays. It is well established that the posttranslationally maturation of proteins, including glycosylation and sialylation processes, vary according to the nature and the level of expression of a large panel of intracellular enzymes. As these processes differ among cell types, they also impact the biological functions and properties of HA and NA, including receptor binding, membrane fusion and antigenicity (for reviews [25], [27], [28]). Thus, we cannot exclude that differences between HeLa and 293-T cells could lead to the expression of HA and NA proteins that differ in their biochemical and biological properties. Although human influenza virus NA displays weak selectivity for different sialic acids [29], subtle differences in the capacity of NA to desialylate HA produced in different cell types cannot be excluded. Finally, the differences between the two assays could relate to the nature of target cells used in each system, i.e. MDCK cells in the fusion assay and MDCK-SIAT1 in the pseudotype infectivity assay. The efficiency of influenza HA-mediated fusion depends on the density of its appropriate receptor on the surface of target cells. In this regard, MDCK-SIAT1 cells express higher level of sialic acid linked to galactose or N-acetylgalactosamine residues by α2,6 bonds [30], which are preferentially used by human influenza HA, as opposed to α2,3 linkages. Thus, higher avidity of HA for α2,6 linked sialic acid residues could explain why smaller amounts of NA are sufficient to reach maximal enhancement of infectivity in MDCK-SIAT1 cells.

## Materials and Methods

### Cell lines

The human cell lines 293-T (ATCC, CRL-11268™) and HeLa (ATCC, CCL-2™) were propagated in Dulbecco's modified Eagle's medium (Gibco) supplemented with 100 U/mL penicillin, 100 µg/mL streptomycin, and 10% fetal calf serum, and the Madin-Darby canine kidney cell lines MDCK-LTR-*lacZ* and MDCK-SIAT1 were propagated in Minimum Essential Medium (Gibco) supplemented with 100 U/mL penicillin, 100 µg/mL streptomycin, and 5% fetal calf serum. The culture medium of the MDCK-SIAT1 cells (Health Protection Agency Culture Collections, catalogue number: 05071502), which were kindly provided by Mikhail Matrosovich (University of Marburg, Marburg, Germany), was further supplemented with 1 mg/mL G418 sulfate (Geneticin; Invitrogen). The MDCK-LTR-*lacZ* cell line was obtained after transfection of the MDCK (ATCC, CCL-34™) cells with a pBlue-TOPO plasmid carrying the HIV-1 LAI LTR (HxB2 nucleotides, nt, 1 to 682) that drives the expression of *lacZ*, and also expressing neomycin resistance (a gift from Sentob Saragosti, Inserm U941, Paris, France). The culture medium of the MDCK-LTR-*lacZ* cell line was supplemented with 2 mg/mL of G418 sulfate. Cells were cultured at 37°C in 5% C0_2_ incubator.

### Construction of plasmids co-expressing influenza HA and HIV-1 Tat

We used the pVITRO2-blasti-mcs plasmid (InvivoGen) that contains two multiple cloning sites (MCS) and allows the silmutaneous coexpression of two genes of interest. The first coding exon of the HIV-1 *tat* gene (HxB2 nt 5,831 to 6,049), encoding aminoacids 1 to 72 and which fully transactivates HIV-1 LTR-driven transcription, was cloned into the first MCS. This exon was amplified by PCR from infectious HIV-1 proviral clone pNL4-3 using primers containing *Cla*I and *Avr*II restriction sites located upstream and downstream of the coding region, respectively, for further cloning. The pVITRO2-blasti-mcs plasmid containing the gene encoding Tat, pVITRO2-blasti-mcs-*tat*, was considered as an acceptor vector for the cloning of various genes encoding HA proteins of interest into the second MCS by using the In-Fusion 2.0 PCR cloning kit (Clontech Laboratories, Inc). In brief, we amplified the HA gene with primers containing *Bgl*II and *Nhe*I restriction sites located on both sides of the open reading frame and that had at least 15 bases of homology with sequences flanking the regions upstream the *Bgl*II site and downstream the *Nhe*I site, respectively, of the pVITRO2-blasti-mcs-*tat* plasmid. After digestion, the amplified fragment was cloned by homologous recombination into the plasmid previously linearized with *Bgl*II and *Nhe*I. This technical approach was used to clone the HA genes of the human H1N1 A/Paris/0650/04 (Accession Number EU685784) and of a highly pathogenic avian (HPA) H7N4 A/Chicken/New South Wales/1/97 (Accession Number AY943924) influenza strains from RNA samples. cDNAs were amplified first from total viral RNA using Superscript Reverse transcriptase (Invitrogen) and the universal primer method described by Hoffmann and al. [31]. PCR reactions were then performed with High Fidelity Taq-DNA polymerase (Invitrogen), and amplified fragments were verified by agarose gel electrophoresis and column-purified (Qiagen) prior to cloning (sequences are available upon request).

### Construction of plasmids expressing influenza NA

The pCI-N1 plasmids encoding wild-type NA from the H1N1 A/Paris/0650/04 virus strain (Accession Number EU718491) was previously described [32]. A defective variant of the NA protein carrying the Y406F mutation (N2 numbering) into the active site of the enzyme was obtained by site-directed mutagenesis using a QuickChange site-directed mutagenesis kit (Stratagene). The construct was verified by sequencing. The other plasmid encoding the N4 protein of the HPA H7N4 influenza stain (Accession Number CY022695) was obtained after amplification of the gene encoding NA from total viral RNA as described above, PCR reaction by using primers containing *Xho*I and *Not*I restriction sites on both sides of the gene, digestion of the amplified fragment, and its cloning between the corresponding restriction sites of the pCI vector.

### Cell-cell fusion assay

HeLa cells transiently coexpressing influenza envelope glycoproteins and HIV-1 Tat were cocultivated with MDCK target cells expressing influenza virus receptors and seeded in microplates at a 1∶0.8 ratio (Figure 1A). Membrane fusion was then quantified using a reporter system based on HIV-1 Tat-dependent expression of β-galactosidase (*lacZ*) [33], [34]. After membrane fusion and cytoplasmic exchanges, the cytosoluble Tat protein induced the synthesis of β-galactosidase, which was measured enzymatically after cell lysis using a colorimetric assay.

The assay was conducted as follows: HeLa cells were seeded into 96-well plates at a density of 10^4^ cells per well and transfected eight hours later by using jetPEI™ transfection reagent (PolyPlus transfection). Cells were transfected with 0.05 µg of pVITRO2-blasti-mcs vector co-expressing the HA and the HIV-1 Tat transactivator proteins, in the absence or in the presence of different quantities of pCI plasmid encoding the influenza NA protein (0.025, 0.05, 0.1, 0.15, or 0.2 µg), and with appropriate amounts of empty pCI vector used as carrier plasmid DNA. All transfections were carried out in triplicate. After 16 hours, the supernatant medium was removed, cells were washed twice with 1X phosphate-buffered saline (PBS), and co-cultured with MDCK-LTR-*lacZ* target cells at a density of 8 10^3^ cells per well. To induce cleavage of the HA0 precursor into mature proteins HA1 and HA2, the coculture medium was removed 24 hours after the start of the coculture and replaced by medium containing trypsin treated with L-(tosylamido-2-phenyl) ethyl chloromethyl ketone (TPCK-treated trypsin, Worthington Biochemical Corporation) at a final concentration of 5 µg/mL. After 15 min at 37°C, this medium was aspirated, and fresh medium was added. Acidic treatment was performed one hour later by replacing the culture medium with DMEM acidified with citric acid (Sigma-Aldrich) at pH 5.0 for 15 min. Medium was further aspirated and replaced by complete medium. Forty hours later, cells were lysed using 100 µl per well of lysis buffer (5 mM MgCl2 and 0.1% NP-40 in 1X PBS), and 100 µl per well of a β-galactosidase chromogenic substrate (6 mM chlorophenol red-β-D-galactopyranoside; CPRG; Roche). Each plate contained a threshold control corresponding to the background noise due to the artefactual transactivation of the LTR by Tat after transfection of the pVITRO2-blasti-mcs-*tat* alone, and a negative control resulting from co-culture between non-transfected HeLa cells and target cells. The optical density (OD) at 575 nm was measured after 1 to 4 hours of incubation at 37°C, and a mean OD value was calculated for each triplicate. The fusion level was calculated as a ratio between the mean OD value obtained for each sample and that of the threshold control, and was considered positive when greater than 1.5.

### Quantification of HA protein in HeLa cells by western blot analysis

HeLa cells seeded into 6-well plates were cotransfected with the H1-expressing plasmid in the absence or in the presence of different quantities of N1-encoding plasmid. The amounts of plasmids were proportional to those used to perform the cell-cell fusion assay, according to the manufacturer's instructions. 40 hours after transfection, cells were harvested in 1 mM PBS-EDTA, washed in 1X PBS, pelleted and resuspended in 300 µl of lysis buffer (50 mM of Tris-HCl [pH 7.8], 150 mM NaCl, 1% NP-40 and 1% protease inhibitor cocktail (P8340, Sigma)). Lysates were cleared by centrifugation (13,000 g; 10 min at 4°C). Cellular proteins were separated by electrophoresis into 10% SDS-PAGE gels, and transferred to a PROTRAN BA 85 membrane (10402588, Whatman GmbH). The blots were probed sequentially with a rabbit anti-H1 antibody (1∶1,000 dilution, 5235, ProSci Incorporated) and a donkey anti-rabbit IRDye 800CW antibody (1∶10,000 dilution; 926-32213, LI-COR Biosciences). The blots were scanned using an Odyssey Infrared imaging system, and fluorescence intensity was analysed using Odyssey application software (version 2.1, Li-Cor Biosciences). The same membrane was washed and further sequentially probed with rabbit anti-actin antibody (1∶400 dilution; A2066, Sigma) and donkey anti-rabbit IRDye 800CW antibody (1∶10,000 dilution). The blots were scanned again and quantified with the Licor software as specified by the manufacturer. The results presented are representative of two independent experiments.

### Indirect evaluation of NA expression on HeLa cells by quantification of the NA enzymatic activity

HeLa cells seeded into 96-well plates were cotransfected as described in the cell-cell fusion assay. 40 hours later, HeLa cells were washed with 1X PBS, and incubated with a high concentration of the fluorogenic substrate 2′-(4 methylumbelliferyl)-α-D-*N*-acetylneuraminic acid (MUNANA) (final concentration of 100 µM) (M8639, Sigma) diluted in MES buffer, as described previously [32]. Fluorescence intensity was then monitored every 10 min (excitation wavelength, 355 nm; and emission wavelength, 460 nm; respectively) by using a Varioskan Flash instrument (Thermo Scientific, Waltham, MA) and Thermo Scientific Skanlt Software (version 2.4.3, Thermo Scientific, Waltham, MA). Each sample was evaluated in triplicate and a negative control corresponding to the fluorescence intensity obtained with HeLa cells transfected with pVITRO2-blasti-mcs-*tat*. The NA enzymatic activity was defined as the slope [(Fluorescence)/(time)] calculated by linear regression using PRISM 4.0b Software.

### *In situ* X-Gal staining of cocultures

Sixty hours after coculture between the transfected HeLa cells and the MDCK-LTR-*lacZ* target cells, adherent cells were fixed in 0.5% glutaraldehyde 20 min at room temperature and stained with the β-galactosidase substrate X-Gal (5-bromo-4-chloro-3-indolyl-beta-D-galactopyranoside; Invitrogen), as described previously [33]. Blue-stained foci were scored under X4 magnification using A Nikon Eclipse TE300 inverted epi-fluoresence microscope.

### Construction of an *env-*defective HIV-1 proviral clone encoding *Renilla* luciferase

The virion infectivity assay used in this study (Figure 1B) is based on the production of HIV-1-like particles expressing influenza A HA and NA proteins, and carrying an HIV-derived vector genome expressing *Renilla* luciferase in place of the HIV-1 *nef* gene. The pNL4-3-ΔENV-LucR vector HIV-1 genome was derived from the pNL4-3 proviral clone in which the *env* gene had been deleted of an internal *Bgl*II fragment corresponding to nucleotides 7,032 to 7,612. To allow the expression of *Renilla* luciferase (LucR) in place of Nef, the *BamH*I (nt 8,462) - *Nco*I (nt 10,567) fragment was removed, and the remaining fragment was ligated to the *BamH*I–*Kpn*I fragment from pTN7-NL [35] and to the *Kpn*I–*Nco*I fragment from pNL4-3.

### Production of HIV-1 particles expressing influenza A HA and NA

Subconfluent monolayer of adherent human 293-T cells seeded in 6-well plates were cotransfected with 0.7 µg of the pNL4-3-ΔENV-LucR vector and 0.5 µg of the pVITRO2-blasti-mcs-*tat* encoding the HA protein from the human H1N1 A/Paris/0650/04 influenza strain, in the absence or in the presence of different amounts of the pCI plasmid encoding the homologous N1 potrein (0.005, 0.05, 0.5, 1, 1.5, or 2 µg), and with appropriate amounts of the empty pCI vector used as carrier plasmid DNA to normalize the quantity of DNA transfected per well by using jetPEI™ transfection reagent (PolyPlus transfection). After 16 hours, cells were washed with 1X PBS and fresh complete culture medium was added, which could be supplemented with exogenous neuraminidase from *Clostridium perfringens* (Worthington Biochemical Corporation) at a final concentration of 15 mU/mL. 24 hours later, supernatant was recovered and centrifuged for 10 min at 2,500 rpm to remove cell debris. To induce the cleavage of the polyprotein precursor HA0 into HA1 and HA2, each viral stock was treated with TPCK-treated trypsin at a final concentration of 15 µg/mL for one hour at 37°C, and HIV-1 p24 antigen content was measured by enzyme-linked immunosorbent assay (ELISA, Innogenetics/Ingen) prior to ultracentrifugation or to infection. To evaluate the potential impact of residual exogenous sialidase activity present in the viral supernatants on infectivity, we ultracentrifugated viral stocks to remove the soluble neuraminidase. Thus, culture supernantants were overlaid on a 20% sucrose cushion (Sigma-Aldrich) in 3.2 mL polycarbonate tubes (Beckman) and ultracentrifugated (TLA 100.4 rotor; Optima™ TLX ultracentifuge; Beckman Instruments) at 45,000 rpm for 90 min at 4°C. Each viral pellet was resuspended with 0.4 mL of DMEM. An aliquot was removed for HIV-1 p24 antigen determination by ELISA prior to measure the infectivity of the normalized resuspended pellets.

### Infectivity assay

Pseudovirus suspensions produced as described above were normalized for HIV-1 p24 content, treated with TPCK-treated trypsin to allow cleavage of the HA0 precursor into the mature proteins HA1 and HA2, and used to infect MDCK-SIAT1 target cells (Figure 1B). Infectivity was determined by measuring luciferase activity in MDCK-SIAT1 lysates. Briefly, 8 10^3^ target cells/well target cells were plated in black-wall, clear bottom 96-well plates (Greiner) 24 h prior to infection. Medium was removed and replaced with 200 µL of complete medium containing serial two-fold dilutions of freshly harvested supernatants from 100 ng p24/mL. Forty hours after infection, 50 µL of 5X luciferase lysis buffer (*Renilla* luciferase assay system, Promega) was added to each well, and plates were maintained at room temperature for 30 min. Wells were sequentially injected with 100 µL of luciferase substrate (Promega), and 3 seconds later, light emission (relative light units, RLU) was measured over a five seconds interval using a Microlumat LB96P luminometer (Berthold). Each sample was evaluated in triplicate, and each experiment contained a negative control corresponding to the RLU values obtained with empty HIV-1 pseudoparticles. The mean RLU value of the negative control was substracted from the mean RLU value of each test sample. RLU were then plotted as a function of amount of HIV-1 p24 used to infect the cells, and infectivity was defined as the slope [(RLU/3 sec)/(ng p24/mL)] as determined by linear regression; in this analysis, each replicate RLU value was considered as an individual point.

### Quantification of HA and NA proteins incorporated into HIV-1 pseudoparticles by western blotting analysis

The amounts of HA and NA proteins into purified virion preparations were quantified by western blot analysis as an estimate of their relative amounts of incorporation into HIV pseudoparticles. 293-T cells were cotransfected in 6-well plates as previously described in the infectivity assay. 40 hours later, particles were recovered from clarified medium by centrifugation for 90 min at 45,000 rpm through a 20% sucrose cushion in a TLA 100.4 rotor. Each viral pellet was resuspended with 120 µl of 10X lysis buffer (200 mM Hepes [pH 7.4], 1.5 M NaCl, 10 mM EDTA, 10% NP-40, and 1% protease inhibitor cocktail (P8340, Sigma, St. Louis)). After quantification of the HIV-1 p24 antigen content, proteins corresponding to 25 ng of p24 were separated by electrophoresis into 10% SDS-PAGE gels, and transferred to a PROTRAN BA 79 membrane (10402088, Whatman GmbH). Proteins reacted with a mixture of rabbit anti-H1 antibody (1∶1,000 dilution, 5235, ProSci Incorporated) and anti-HIV-1 p24 antibody (1∶1,000; ab9071, Abcam) followed by a mixture of donkey anti-rabbit IRDye 800CW antibody (1∶10,000 dilution; 926-32213, LI-COR Biosciences) and donkey anti-mouse IRDye680CW antibody (1∶10,000 dilution; 926-32222, LI-COR Biosciences). The blots were scanned using an Odyssey Infrared imaging system, and fluorescence intensity was analysed using Odyssey application software (version 2.1, Li-Cor Biosciences). The same membrane was further incubated with a rabbit anti-N1 antibody (1∶500 dilution, 5245, ProSci Incorporated) and then with donkey anti-rabbit IRDye 800CW antibody (1∶10,000 dilution).
